# From Bench to Bedside: A Team’s Approach to Multidisciplinary Strategies to Combat Therapeutic Resistance in Head and Neck Squamous Cell Carcinoma

**DOI:** 10.3390/jcm13206036

**Published:** 2024-10-10

**Authors:** Bridget E. Crossman, Regan L. Harmon, Kourtney L. Kostecki, Nellie K. McDaniel, Mari Iida, Luke W. Corday, Christine E. Glitchev, Madisen T. Crow, Madelyn A. Harris, Candie Y. Lin, Jillian M. Adams, Colin A. Longhurst, Kwangok P. Nickel, Irene M. Ong, Roxana A. Alexandridis, Menggang Yu, David T. Yang, Rong Hu, Zachary S. Morris, Gregory K. Hartig, Tiffany A. Glazer, Sravani Ramisetty, Prakash Kulkarni, Ravi Salgia, Randall J. Kimple, Justine Y. Bruce, Paul M. Harari, Deric L. Wheeler

**Affiliations:** 1 Department of Human Oncology, University of Wisconsin, Madison, WI 53705, USA; bridget.crossman@wisc.edu (B.E.C.); rlharmon2@wisc.edu (R.L.H.); iida@humonc.wisc.edu (M.I.); maharris7@wisc.edu (M.A.H.); clin399@wisc.edu (C.Y.L.); kpnickel@humonc.wisc.edu (K.P.N.); zmorris@humonc.wisc.edu (Z.S.M.); rkimple@humonc.wisc.edu (R.J.K.); harari@humonc.wisc.edu (P.M.H.); 2 Departments of Biostatistics and Medical Informatics, University of Wisconsin, Madison, WI 53726, USA; clonghurst@wisc.edu (C.A.L.); irene.ong@wisc.edu (I.M.O.); alexandridis@biostat.wisc.edu (R.A.A.); 3 Carbone Cancer Center, University of Wisconsin, Madison, WI 53792, USA; rhu@uwhealth.org (R.H.); jybruce@medicine.wisc.edu (J.Y.B.); 4 Department of Obstetrics and Gynecology, University of Wisconsin, Madison, WI 53705, USA; 5 Department of Biostatistics, University of Michigan, Ann Arbor, MI 48109, USA; menggang@umich.edu; 6 Department of Pathology and Laboratory Medicine, University of Wisconsin, Madison, WI 53705, USA; dtyang@wisc.edu; 7 Department of Surgery, University of Wisconsin, Madison, WI 53705, USA; hartig@surgery.wisc.edu (G.K.H.); glazer@surgery.wisc.edu (T.A.G.); 8 Department of Medical Oncology and Therapeutics Research, City of Hope Comprehensive Cancer Center, Duarte, CA 91010, USA; sramisetty@coh.org (S.R.); pkulkarni@coh.org (P.K.); rsalgia@coh.org (R.S.); 9 Department of Medicine, University of Wisconsin, Madison, WI 53705, USA

**Keywords:** HNSCC, EGFR, resistance, cetuximab, TAMs, Axl, MerTK

## Abstract

Head and neck squamous cell carcinoma (HNSCC) is diagnosed in more than 71,000 patients each year in the United States, with nearly 16,000 associated deaths. One significant hurdle in the treatment of HNSCC is acquired and intrinsic resistance to existing therapeutic agents. Over the past several decades, the University of Wisconsin has formed a multidisciplinary team to move basic scientific discovery along the translational spectrum to impact the lives of HNSCC patients. In this review, we outline key discoveries made throughout the years at the University of Wisconsin to deepen our understanding of therapeutic resistance in HNSCC and how a strong, interdisciplinary team can make significant advances toward improving the lives of these patients by combatting resistance to established therapeutic modalities. We are profoundly grateful to the many scientific teams worldwide whose groundbreaking discoveries, alongside evolving clinical paradigms in head and neck oncology, have been instrumental in making our work possible.

## 1. Introduction

Head and neck squamous cell carcinoma (HNSCC) arises from the soft mucosal tissue of the head and neck area, including the oral cavity, oropharynx, larynx, and pharynx [1]. HNSCC accounts for 4.5% of all cancer diagnoses around the world. In the United States alone, more than 71,000 new cases of HNSCC are diagnosed each year, with approximately 16,000 associated deaths [2]. The disease is associated with tobacco and alcohol use, as well as human papillomavirus (HPV) infection. While mortality has slowed in recent years, the improved survival rate is modest and likely due to the declined use of tobacco products and the rise in HPV-positive disease, which has a favorable prognosis [1,2]. Early treatment strategies for HNSCC patients revolved around surgery and radiation therapy. However, the rapidly proliferating nature of HNSCC posed a significant barrier to optimizing tumor control with radiotherapy alone [3,4,5]. In 1999, at the University of Wisconsin, Dr. Paul Harari’s group identified that targeting the epidermal growth factor receptor (EGFR) with the monoclonal antibody cetuximab (C225) resulted in reduced proliferation, cell cycle arrest, enhanced apoptosis, and improved radiosensitivity of HNSCC cells in vitro [6]. Furthermore, the Harari lab reported that EGFR blockade with cetuximab reduced HNSCC cell migration and invasion in vitro and in vivo [7]. The finding that EGFR blockade enhanced the effects of radiation therapy in HNSCC in preclinical studies contributed to the landmark randomized phase 3 clinical trial reporting enhanced survival and locoregional control when cetuximab was administered with radiation [8,9,10]. As a result, cetuximab, combined with radiotherapy, was approved by the FDA for treating locoregionally advanced HNSCC in 2006. Several years thereafter, the RTOG 1016 trial aimed to determine whether the replacement of cisplatin with cetuximab could preserve high survival but reduce treatment-related toxicity in patients with HPV-positive HNSCC. The results from this randomized, multicenter, non-inferiority trial indicated that in HPV-positive oropharyngeal carcinoma, radiotherapy plus cetuximab was inferior to radiotherapy plus cisplatin in terms of overall survival, thereby maintaining radiotherapy plus cisplatin as the standard of care for this population [11]. Multiple clinical trials remain in progress to assess various treatment de-intensification strategies for the HPV-positive population to diminish long-term toxicity profiles while preserving high survival rates.

**Figure 1:**
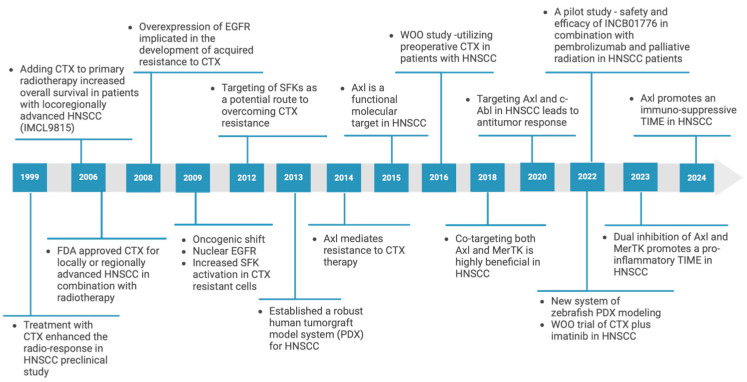
University of Wisconsin/City of Hope team: discoveries through the years.

A major persistent challenge in head and neck oncology involves improving treatment outcomes for patients with HPV-negative disease who often develop resistance to therapy and demonstrate high recurrence rates following treatment. One such challenge is acquired resistance to therapeutic modalities. While 36% of patients initially respond to the EGFR inhibitor cetuximab when given in combination with doublet cytotoxic chemotherapy, they experience a high rate of disease recurrence. Beyond acquired resistance, many patients also exhibit intrinsic resistance, wherein no tumor regression is observed following first-line chemotherapy or radiation [12]. Receptor tyrosine kinases (RTKs) are critical mediators in intrinsic and acquired resistance to cetuximab in HNSCC and represent viable therapeutic targets to overcome resistance. The Wheeler lab began in 2008 at the University of Wisconsin and has focused on uncovering mechanisms of therapeutic resistance in HNSCC, leading to several foundation and federal grants, including major projects in two NCI-funded Specialized Program of Research Excellence (SPORE) grants. The cornerstone of our team’s successes in driving translational science from the bench to bedside via clinical trials is a broad collaboration among an interdisciplinary team including basic and translational scientists, clinicians, surgeons, bioinformaticians, statisticians, and pathologists at the University of Wisconsin and beyond (Figure 1).

## 2. Cetuximab Resistance in HNSCC

### 2.1. HER Family Receptor Tyrosine Kinases as a Mediator of Acquired Resistance to Cetuximab

The epidermal growth factor receptor (EGFR) is an RTK whose overexpression has been implicated in many solid cancers, including HNSCC, lung, pancreas, and colorectal cancers [13,14]. The EGFR belongs to a family of RTKs, which include HER2, HER3, and HER4 [15]. Activation of EGFR is well known to stimulate downstream signaling pathways, including MAPK and PI (3)K/Akt, which are involved in the regulation of cell proliferation, survival, angiogenesis, and metastasis, making it an enticing antitumor target [16]. One major hurdle in treating HNSCC patients with cetuximab is acquired resistance [17,18]. While critical mutations in EGFR had previously been linked to resistance to small-molecule inhibitors, the mechanism for acquired resistance to cetuximab remained unclear in the early 2000s [19,20]. In 2008, our group hypothesized that blockade of EGFR activation with cetuximab would result in the activation of other RTKs (oncogenic shift) with similar activity to EGFR, leading to acquired resistance. In a study published in Oncogene, we identified that cells chronically challenged with cetuximab, ligand-bound EGFR, and other HER family members did not undergo typical lysosomal degradation due to a lack of c-Cbl ubiquitination, leading to enhanced HER family expression in cells with acquired resistance. Consequently, EGFR was engaged in the transphosphorylation of three RTKs with similar signaling modalities: HER2, HER3, and cMet. Upon further probing the roles of HER2, HER3, and cMet in resistance to cetuximab, cMet activation was found unlikely to be a contributing factor, as knockdown did not reduce the proliferative potential of resistant cells treated with cetuximab. Conversely, it was identified that administering cetuximab and 2C4, a monoclonal antibody preventing the heterodimerization of HER2 and HER3, promoted the suppression of HER3 and PI(3)K/Akt activation, which correlated with a robust anti-proliferative signature. The knockdown of HER3 also resensitized resistant cells to cetuximab, indicating that HER3 may represent a key node in acquired resistance to cetuximab treatment in HNSCC [21]. 

### 2.2. Targeting Src Family Kinases to Overcome Cetuximab Resistance

Building on the work that identified the overexpression of HER family receptor tyrosine kinases as a mediator of acquired resistance to cetuximab, we identified robust phosphorylation of three tyrosine residues, Y1173, Y1101, and Y845, on the cytoplasmic tail of the EGFR in several cetuximab-resistant cell models. While Y1173 is an autophosphorylation site, Y845 and Y1101 are phosphorylated by Src family kinases (SFKs) [22], suggesting that these three tyrosine residues may represent three critical nodes that, in part, lead to cetuximab resistance. Experiments designed to target EGFR activity using erlotinib or dasatinib to target EGFR and SFKs could resensitize cetuximab-resistant models. This work implicated specific tyrosines on the c-terminal tail of EGFR in manifesting cetuximab resistance. Studies designed to look downstream of the SFK phosphorylation sites on EGFR indicated that the inactivation of SFKs by dasatinib led to decreased phosphorylation of HER3 and, thus, decreased PI(3)K/Akt activation in cetuximab-resistant cell clones, indicating that HER3 played a crucial role in resistance. Indeed, administration of dasatinib alone and dasatinib in combination with cetuximab decreased cell proliferation and reduced the phosphorylation of HER3 and Akt. Given these results, the dual targeting of EGFR and SFKs represents a potential therapeutic strategy to overcome acquired resistance to cetuximab in the clinic [23].

### 2.3. SFK Mediates Nuclear Translocation of EGFR to Promote Cetuximab Resistance

The shuttling of EGFR from the cytoplasm to the nucleus has been widely observed in many cancer types and is often associated with poor clinical outcomes [24,25,26,27,28,29,30,31,32,33,34,35,36,37]. With previous studies out of our lab suggesting that EGFR and SFKs are critical mediators in acquired resistance to cetuximab [21,23], we hypothesized that increased SFK activity might be linked to EGFR translocation to the nucleus (nEGFR). In a 2009 study published in Oncogene, we identified robust nEGFR expression in cetuximab-resistant cell clones relative to their cetuximab-sensitive parental cells [38]. Furthermore, we observed that the ligand for EGFR, EGF, is present at increased levels in cetuximab-resistant cell clones, contributing to the chronic translocation of EGFR to the nucleus. Blocking the release of EGF in resistant cells restored EGFR’s localization to the cytoplasm, and the addition of conditioned media from resistant cells conferred resistance to cetuximab in sensitive cells. 

When treated with dasatinib, robust decreases in nEGFR and increases in membrane-bound EGFR were observed in a series of cetuximab-resistant clones. Dual treatment of these cells with dasatinib and cetuximab restored the sensitivity of cells resistant to cetuximab treatment. In an in vivo model, tagging EGFR with a nuclear localization sequence to sequester EGFR to the nucleus conferred resistance to cetuximab in athymic nude mice [38]. Mechanistically, a series of point mutations in the EGFR c-terminus were used to identify that SFKs phosphorylate EGFR at Y1101, and this phosphorylation is critical for shuttling EGFR to the nucleus [39]. In a later study, we reported similar results in HNSCC, wherein nEGFR, caused either by cetuximab [40] or radiation treatment [41], conferred resistance to both treatment modalities and that therapeutic sensitivity can be restored by blocking SFK signaling with dasatinib [42]. This work was broadened beyond HNSCC, where we identified a role for nEGFR in triple-negative breast cancer (TNBC) [43]. It is well established that EGFR is overexpressed in TNBC, but cetuximab therapy shows little clinical activity in this setting [44,45]. We postulated that nuclear accumulation and the function of EGFR may be responsible. Leaning on lessons from cetuximab-resistant models in HNSCC, we identified that blockade of SFK activity resulted in decreased Y1101 phosphorylation and accumulation of EGFR on the cellular membrane. This approach decoupled the cytoplasmic and nuclear signaling components of EGFR and forced the cell to rely solely on classical membrane signaling, which could be abolished by cetuximab. This finding led to federal funding to support mechanistic studies and a proof-of-principle clinical trial in women with TNBC. 

### 2.4. Axl as a Critical Contributor to Cetuximab Resistance

The TAM family of RTKs includes three receptors: Tyro3, Axl, and MerTK. This family of receptors has been implicated in the progression and metastasis of several types of tumors, both blood and solid cancers [46,47,48,49,50,51,52,53,54,55,56]. Furthermore, the family member Axl has been linked to therapeutic resistance, particularly to tyrosine kinase inhibitors targeting EGFR [57,58,59,60]. We found Axl to be overexpressed and phosphorylated in cells that acquire resistance to cetuximab. In addition, in experiments aimed at knocking down Axl in resistant clones, cell proliferation, EGFR phosphorylation, downstream MAPK, and AKT activation decreased significantly. In a reciprocal study knocking down EGFR, similar results were observed in diminished total Axl levels and downstream signaling. Furthermore, co-immunoprecipitation studies revealed the association of Axl and EGFR in cetuximab-resistant cell clones but not the parental line. Additionally, cetuximab-resistant clones were found to be sensitive to the anti-Axl monoclonal antibody Mab173 and the Axl-targeting tyrosine kinase inhibitor (TKI) bemcentinib, with robust decreases in proliferation, EGFR protein levels, and MAPK signaling observed. We performed confirmatory studies, overexpressing Axl in cetuximab-sensitive cell lines, which resulted in robust EGFR and MAPK phosphorylation and resistance to cetuximab compared with parental cells. Similar results were observed in in vivo murine studies, where tumors that developed acquired resistance to cetuximab had increased levels of Axl phosphorylation at tyrosine 779. Furthermore, in patient-derived xenograft models, cetuximab-sensitive tumors exhibited low Axl levels by immunohistochemistry (IHC) staining, while tumors intrinsically resistant to cetuximab had increased total and phosphorylated Axl expression levels. In a 2009 study published in Oncogene, we identified the upregulation of EGFR ligands in the context of cetuximab resistance. The study found that stimulation with either EGF or TGFα increased Axl phosphorylation and association with EGFR, indicating that EGFR ligands may lead to cetuximab resistance by activating Axl and promoting association with EGFR. While initial studies were performed in a cetuximab-resistant model of non-small cell lung cancer (NSCLC), similar results were observed in models of HNSCC, indicating a more global role of Axl-EGFR-mediated resistance to cetuximab in solid tumors [61].

### 2.5. Patient-Derived Model’s Role in Studying Therapeutic Resistance in HNSCC

Coincident with the work described above, Dr. Randall Kimple was recruited to the University of Wisconsin. The Kimple lab was instrumental in establishing some of the initial patient-derived model systems utilized by our group to study therapeutic resistance in HNSCC [62,63,64,65]. These models were initially developed from surgical specimens; thus, the team could not know how the patient would have responded to a given treatment. This limitation has been overcome in ongoing window-of-opportunity (WOO) studies in which pre-chemotherapy biopsies are used to establish patient-derived xenograft (PDX) models. Ongoing NIH-funded work seeks to expand our understanding of PDX models further, including how faithfully they replicate patient responses, the impact of orthotopic modeling, and how best to integrate an immune system into the modeling. The development of PDX model systems allowed for integration between the Wheeler and Kimple laboratories, which helped test several mechanistic findings centered on cetuximab resistance. 

Expanding on model systems, Drs. Wheeler and Kimple integrated with the Salgia lab from the City of Hope Comprehensive Cancer Center. Here, the team focused on utilizing zebrafish to predict patient responses to therapy. Dr. Salgia’s newly designed system of PDX modeling in zebrafish is being used to test and correlate tumor responses. This system is faster, more cost-efficient, and more stable than mouse PDX modeling and will significantly improve the time it takes to evaluate the response of patient tumors to targeted therapeutics, leading to a rapid next-generation personalized medicine approach. 

### 2.6. Targeting Axl to Overcome Therapeutic Resistance in HNSCC

The work of our HNSCC research team, led by Dr. Harari at the University of Wisconsin, led to a Specialized Program in Research Excellence (SPORE) grant, awarded in 2016, the first to be awarded at the University of Wisconsin. This NIH National Institute for Dental and Craniofacial Research (NIDCR) and National Cancer Institute (NCI)-funded award is the pinnacle of collaboration and teamwork, allowing four independent research projects led by translational scientists and clinicians, three supportive cores, a group of patient advocates, and early-career investigators to work in unison toward improved clinical outcomes for HNSCC patients. One project that contributed to the inception of the UW HNSCC SPORE program was led by Drs. Wheeler and Kimple focused on targeting Axl to overcome resistance to cetuximab. In this study, we found that treatment with the Axl-targeting bemcentinib reduced HNSCC cell proliferation, invasion, and migration and increased the sensitivity of cells to cetuximab, chemotherapy, and radiation [66]. 

### 2.7. Blockade of Abl Overcomes Resistance to Cetuximab

Building off of our 2014 study published in Cancer Research, we confirmed the role of Axl in mediating resistance to cetuximab in PDX models of HNSCC in our 2020 study published in Clinical Cancer Research [67]. To further understand the mechanism by which Axl mediates cetuximab resistance, we mutated three critical tyrosine residues (Y779, Y821, and Y866) on Axl’s c-terminal domain. We overexpressed these constructs in cetuximab-sensitive HNSCC cells. This study suggested that cells overexpressing Axl wild-type (WT), Y779F, and Y866F constructs remained resistant to cetuximab. In contrast, cells overexpressing the Axl Y821F construct were sensitive to cetuximab in both in vitro and in vivo models of HNSCC. Reverse-phase protein array (RPPA) analysis of vector, Axl-WT, and Axl-Y821F cell lines revealed increased phosphorylation of the c-Abl non-receptor tyrosine kinase in Axl-WT and decreased phosphorylation of c-Abl in Axl-Y821F cells relative to a vector control. To test whether Axl signaling through Y821 to c-Abl was responsible for mediating cetuximab resistance, c-Abl was knocked down in cetuximab-resistant cells, leading to cetuximab sensitivity. Similar results were observed with pharmacological inhibition of c-Abl. In in vivo studies treating tumors expressing Axl-WT with either a vehicle control, cetuximab, imatinib (a TKI targeting c-Abl), or a combination of cetuximab plus imatinib, c-Abl inhibition was able to overcome cetuximab resistance. Similar results were observed in PDX models of cetuximab-resistant HNSCC, where the combination of cetuximab plus imatinib resulted in a significant delay of tumor growth and complete tumor regression in more than 50% of tumors without recurrence. Collectively, these data provided strong evidence that blockade of c-Abl kinase signaling can restore tumor sensitivity to cetuximab. That study, borne from the UW HNSCC SPORE, resulted in a successful R01 submission with the third aim of this grant focused on a WOO trial of cetuximab plus imatinib evaluating the response, safety, and feasibility of treating patients with this combination prior to surgery or radiation to prolong patient lives by overcoming the hurdle of acquired resistance to cetuximab.

## 3. The TAM Family of RTKs Contributes to Progression of HNSCC

### 3.1. Axl and MerTK Compensate for One Another to Drive HNSCC Progression

After implicating Axl in cetuximab resistance, we sought to explore the impact of targeting the TAM receptors in solid tumors, including HNSCC, NSCLC, and TNBC, with the hypothesis that Axl inhibition would impact proliferation, survival, and metastasis. In our study published in Molecular Cancer Therapeutics, we identified an intrinsic and adaptive feedback mechanism leading to resistance to Axl-targeting agents mediated by the upregulation of MerTK [68] when Axl was directly targeted. In this study, we found that human HNSCC cell lines with high expression of MerTK correlated with resistance to anti-Axl therapeutics. Targeting Axl via genetic or molecular inhibition increased MerTK expression in vitro and in vivo. Dual treatment of these cell lines with bemcentinib and UNC2025, a MerTK-directed TKI, significantly inhibited tumor cell expansion in vitro and impacted tumor growth in vivo compared with single inhibition of Axl or MerTK alone. These results highlighted a synergistic relationship between MerTK and Axl in driving HNSCC and provided a rationale for developing therapeutic strategies targeting both receptors to inhibit HNSCC growth and progression [68]. 

### 3.2. Dual Inhibition of Axl and MerTK for Treatment of HNSCC—Collaborating with Industry Partners

To build upon our findings on dual Axl and MerTK inhibition to treat HNSCC, we collaborated with Incyte Pharmaceuticals to obtain a novel kinase inhibitor, INCB08INCB081776 (INCB081776), targeting both Axl and MerTK [69]. INCB081776 is a highly selective, ATP-competitive inhibitor shown to inhibit the kinase activity of Axl and MerTK [70]. In 2023, we published a study in Head & Neck investigating the effects of INCB081776 on HNSCC models in vitro and in vivo [71]. We found that INCB081776 inhibited tumor-bound Axl and MerTK kinase activity and downstream signaling pathway activation in mouse oral cancer (MOC) cell lines. Treatment with INCB081776 also delayed the growth of human HNSCC PDX and MOC cell lines in vivo compared with single inhibition of Axl and MerTK alone. Given the increasing evidence that the TAM family can play an immunomodulatory role in the tumor immune microenvironment (TIME) [72,73,74,75,76,77,78,79], we sought to explore the effects of dual Axl/MerTK inhibition on the TIME. When treated with INCB081776, MOC cell lines had decreased levels of pro-tumor cytokine production (such as TNF-alpha and IL-1beta), increased infiltration of antitumor immune cells (such as cytotoxic T-cells and NK cells), and decreased infiltration of pro-tumor immune cells (such as T regulatory cells and M_2_ macrophages). Additionally, treating tumor-bearing mice with a combination of INCB081776 and anti-PDL1 led to an even more significant delay in tumor growth compared with INCB081776 or anti-PDL1 alone. Overall, it was determined that inhibition of Axl and MerTK by INCB081776 led to a more “antitumor” TIME, ushering in a new era for our team to determine how Axl and MerTK contribute to an immunosuppressive and immunoevasive TIME in HNSCC [71]. 

### 3.3. Targeting Axl and MerTK Simultaneously: Turning a Cold Tumor Immune Microenvironment to Hot—Building a Team to Harness the Immune System to Treat HNSCC Patients

The discovery that targeting tumor-bound Axl and MerTK significantly influenced the TIME in HNSCC led to our involvement in renewing the UW HNSCC SPORE grant in 2021. One of the aims outlined in this resubmission was delineating Axl and MerTK’s mechanistic actions that contribute to an immunosuppressive TIME in HNSCC. Upon the successful renewal of the SPORE grant, we first began investigating Axl’s role in creating a pro-tumor, anti-inflammatory TIME. In a recent experiment examining Axl’s influence on the TIME, CRISPR/Cas9 was used to knock out Axl in the MOC2 cell line, creating three Axl knockout (KO) clones. The growth of Axl KO tumors in vivo was significantly slower compared with parental MOC2; however, IHC analysis showed no change in Ki67 levels in the KO tumors. These results indicated that the change in tumor growth in Axl KOs was not due to changes in the proliferation rate but potentially due to alterations in the TIME’s composition. In a subsequent study, tumor growth was impacted when implanted in immune-competent mouse models, further indicating that the tumor growth delay may be immune-mediated. Co-culture experiments revealed higher lymphocyte cytotoxicity against Axl KO cell clones than parental, and in vivo, lymphocyte depletion studies rescued the growth of the Axl KO cell lines to similar rates as parental. Furthermore, the analysis of cytokine and chemokine production by a variety of methods indicated that Axl expression may alter the secretion of pro-inflammatory cytokines and chemokines, but further analysis is needed to understand this mechanism as well as how MerTK plays an immunomodulatory role in cooperation with Axl.

Exploring the immunomodulatory role of Axl and MerTK in HNSCC patients has yet to be studied, making it a central focus for our team. Due to the success of the dual Axl and MerTK inhibitor, INCB081776, in preclinical HNSCC models, the SPORE resubmission included a pilot study evaluating the safety and efficacy of INCB081776 in combination with anti-PD1 checkpoint blockade (pembrolizumab) and palliative radiation in HNSCC patients. That work formed a robust collaborative team including Drs. Justine Bruce (medical oncologist), Tiffany Glazer and Gregory Hartig (surgeons), Zachary Morris (radiation oncologist/immunologist), Roxana Alexandridis (biostatistician), Irene Ong (bioinformatician), and Rong Hu (pathologist) from the UW Carbone Cancer Center. The clinical trial, which began recruiting patients in June 2024, consists of 12 participants with recurrent or metastatic HNSCC. Upon recruitment and screening, patients will undergo a baseline biopsy from one lesion without previous irradiation or locoregional therapy. Patients will then receive daily oral doses of INCB081776 for 52 days. On days 9–14, patients will undergo a second biopsy before receiving their first dose of intravenous pembrolizumab on day 15. Between days 29 and 33, patients will complete palliative radiation therapy on a non-biopsy lesion, along with another dose of pembrolizumab on day 36. Patients will then undergo a final biopsy between days 37 and 56. There will be three biopsies per patient: one before receiving any of the treatments above, one after receiving only INCB081776, and one after receiving INCB081776 + pembrolizumab + palliative radiation. While the primary and secondary endpoints of this study aim to evaluate the safety and efficacy of this therapeutic regimen, we will have the unique opportunity to determine if the TIME changes throughout treatment, further delineating how Axl and MerTK can play an immunomodulatory role in HNSCC. The evaluation of the TIME will be carried out on biopsy samples using digital spatial profiling technology and multiplex IHC. That clinical trial has resulted from our team’s efforts to translate our findings from the bench to the bedside, leading to the critical step of translating clinical outcomes from the bedside back to the bench.

## 4. The Future of Axl Targeting for Improved Therapeutic Effect

As our team shifts our focus to the immunomodulatory role of Axl and MerTK, the exact signaling mechanism to create an immunosuppressive TIME remains unknown. It is hypothesized that Axl and MerTK are activating downstream pathways that regulate these immunomodulatory effects, which leads to the question that could result in the subsequent clinical trial: what signaling pathways downstream of Axl and MerTK are involved in modulating the TIME, and can targeting them enhance the effects of dual Axl/MerTK inhibition using INCB081776? Previous studies have implied that targeting RTKs along with their downstream signaling pathways can enhance inhibitory effects, so we seek to further target Axl and MerTK in the setting of HNSCC [80,81,82]. The goal is to identify druggable nodes in the downstream signaling pathway of Axl and MerTK that lead to an immunosuppressive TIME and to inhibit those nodes along with Axl and MerTK. For example, if a key downstream pathway signaling is hypothetically found to be contributing to the immunomodulatory effects seen in HNSCC, this could result in preclinical and clinical trials evaluating combination therapy of INCB081776 + targeted inhibitor + pembrolizumab to treat HNSCC patients. We hypothesize that inhibiting Axl and MerTK along with a downstream signaling pathway could lead to a pro-inflammatory TIME and potentially enhance the effects of current and future immunotherapies.

## 5. Discussion

**Figure 2:**
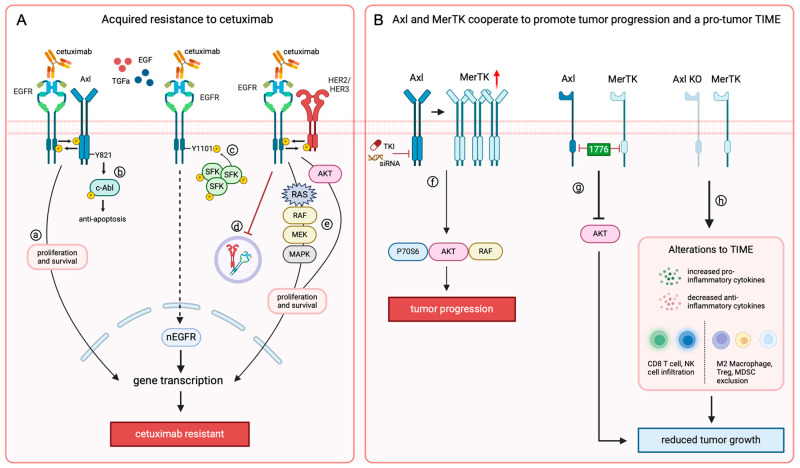
RTKs’ role in therapeutic resistance and HNSCC progression. (**A**) In the context of chronic treatment, HNSCC can acquire resistance to cetuximab when (**a**) transphosphorylation of EGFR and Axl activates proliferation and survival pathways [61,66], (**b**) Axl activates c-Abl signaling through Y821 to inhibit apoptosis [67], (**c**) increased levels of EGF in the cytoplasm lead to SFK-mediated translocation of EGFR to the nucleus (nEGFR) via Y1101 [38,39,42,43], (**d**) decreased lysosomal degradation of EGFR and HER family members leads to persistent membrane expression [21], and (**e**) transphosphorylation of membrane-bound EGFR and HER2 or HER3 leads to activation of RAS, RAF, MEK, MAPK, and AKT pathways to promote tumor cell proliferation and survival. (**B**) Tumor-bound Axl and MerTK have been implicated in promoting tumor progression, and we found that (**f**) targeting Axl alone results in increased MerTK expression, leading to P70S6, RAF, and AKT activation and tumor progression [68], and (**g**) AKT activation can be turned off by targeting both Axl and MerTK with the dual inhibitor INCB081776 [71]. Furthermore, (**h**) genetic knockout of Axl can switch the TIME from cold to hot, leading to reduced tumor growth both in vitro and in vivo.

Translating basic discoveries into clinical trials and improved patient outcomes requires a strong, interdisciplinary investigative team. Mechanistic discoveries in the laboratory are made possible by collaboration among scientists, postdoctoral fellows, students, pathologists, statisticians, and bioinformaticians. To translate these discoveries, investigators must partner with one another as well as clinicians and clinical research coordinators to identify appropriate patient populations and translational modalities to generate the greatest possible outcomes in improving patient care. Our collaborative team has made fundamental discoveries in identifying HER family members as critical mediators of resistance to cetuximab in HNSCC patients and uncovering that targeting c-Abl and Axl can aid in resensitizing patients to cetuximab therapy (Figure 2A). Furthermore, collaboration with colleagues with expertise in tumor immunology, notably, Dr. Zachary Morris, allowed our team to make strides in discovering that Axl and its TAM family member, MerTK, work together to regulate tumor-intrinsic mechanisms of immune invasion and suppression, leading to HNSCC progression (Figure 2B). 

In partnering with the more extensive HNSCC research and clinical community at the University of Wisconsin and the City of Hope, our team contributed to two consecutive HNSCC SPORE awards. The initial SPORE project led by Dr. Wheeler and Dr. Kimple culminated in a window of opportunity (WOO) trial investigating resistance mechanisms in patients treated with cetuximab. The results of this trial led to a subsequent WOO trial funded by an R01 grant for a combination treatment of cetuximab plus imatinib. The present SPORE project, led by Dr. Wheeler and Dr. Bruce, is an evolution into the space of tumor immunology based on the discovery that Axl and MerTK work together to promote a “cold” TIME. Culminating in a clinical trial of the dual inhibition of Axl and MerTK in combination with radiation and anti-PD1 checkpoint blockade in collaboration with Incyte Corporation, this project is a strong example of what can be accomplished when scientific investigators, clinicians, and industrial partners come together with a shared goal of improving outcomes for cancer patients. 

One of the largest challenges in forwarding research toward improved patient outcomes is investigators and clinicians working independently of one another. Without strong collaboration, researchers may not be pushing science toward the bettering of the patients they wish to help, and clinicians may not know what innovations are around the corner. For example, an investigator might make a scientific discovery that, while novel, is not relevant to the patient populations they wish to treat because they have not harnessed tissue or informatics resources that could be available to them or discussed successes and setbacks with investigators in adjacent fields. This can cause significant challenges in driving forward discoveries and chances at longer lives for the patients we undertake this work for. The University of Wisconsin’s research environment has been instrumental in the ability of researchers to build and maintain multidisciplinary relationships for decades. Beyond a full calendar of tumor boards, internal and external seminars, and grand rounds, this rich environment allows investigators and clinicians to work hand in hand with ease, with office, laboratory, pathology, statistical, and bioinformatic resources and clinical spaces all housed in the same building. In this way, investigators, graduate students, and clinicians have readily been able to develop interpersonal relationships and meet frequently to move research forward in a fast-paced, patient-centric manner, with all the resources they need just steps away. Clinicians can identify areas where the patients they are seeing could benefit from new therapeutic strategies and can take that information directly to scientists upstairs, working together to progress basic and translational research into clinical trials. While this ultra-collaborative environment brings diverse perspectives and robust networks together to drive forward new scientific discoveries, the ultimate triumph of the UW HNSCC team has been improving outcomes for cancer patients in our communities every day. The UW team would like to thank all HNSCC researchers and clinicians worldwide who are making fundamental discoveries in the laboratory and advancing these into clinical trials to treat HNSCC patients. The discoveries highlighted in this paper were only possible due to the groundbreaking work of others. We are proud to be part of a global team working to cure HNSCC.
